# Single-nucleus RNA sequencing reveals transcriptional heterogeneity in the blastema of favorable-histology Wilms tumor

**DOI:** 10.1172/jci.insight.188078

**Published:** 2026-06-09

**Authors:** Mike Adam, Keri A. Drake, Naomi Pode-Shakked, Katherine VandenHeuvel, S. Steven Potter, James Geller

**Affiliations:** 1 Division of Experimental Hematology and Cancer Biology, Cancer and Blood Diseases Institute, Cincinnati Children’s Hospital Medical Center, Cincinnati, Ohio, USA.; 2 Division of Pediatric Nephrology, University of Texas Southwestern Medical Center, Dallas, Texas, USA.; 3 Pediatric Nephrology Unit, Dana Dwek Children’s Hospital, Tel Aviv Sourasky University Medical Center (Ichilov), Tel Aviv, Israel.; 4 Grey Faculty of Medical & Health Sciences, Tel Aviv University, Tel Aviv, Israel.; 5 Division of Pathology and Laboratory Medicine, and; 6 Division of Developmental Biology, Cincinnati Children’s Hospital Medical Center, Cincinnati, Ohio, USA.; 7 Division of Hematology/Oncology, Department of Pediatrics, Peckham Center for Cancer and Blood Disorders, Rady Children’s Hospital, San Diego, California, USA.; 8 UCSD, La Jolla, California, USA.

**Keywords:** Development, Nephrology, Oncology, Cancer

## Abstract

While Wilms tumors commonly arise from renal precursor cells and maintain features of the developing kidney, recent studies have demonstrated substantial genetic, histologic, and molecular heterogeneity. To further investigate tumor variability as well as unifying features in tumor biology, we performed single-nucleus RNA sequencing (snRNA-seq) on treatment-naive, favorable-histology Wilms tumors utilizing a reference atlas established from tumor-adjacent kidney samples and fetal kidney. Transcriptional profiles of blastemal, stromal, and epithelial components were correlated with tumor histology and demonstrated developmental-lineage plasticity, with *PAX2* and *PAX8* expression normally restricted to the nephron lineage of the fetal kidney found to be expressed in tumor stroma, as well as the stromal marker *POSTN* identified in tumor blastema. Further analyses of the blastema show shared transcriptional features with the differentiation trajectory of “uninduced” to “early differentiating” fetal nephron progenitor cells as well as aberrant expression of stromal signatures. A number of pathways from fetal nephron progenitors were maintained in the blastema, including regulation of stem cell maintenance and axonogenesis, whereas other pathways appear enriched in specific tumor samples, demonstrating the ability of snRNA-seq to identify both unifying transcriptional signatures and uncover distinct molecular targets in signaling pathways and/or biological drivers of Wilms tumorigenesis.

## Abstract 2

Wilms tumors commonly arise from and maintain features of the developing kidney, this embryonal tumor has been shown to exhibit surprisingly heterogeneous genetic, histological, and molecular landscapes.

## Graphical abstract

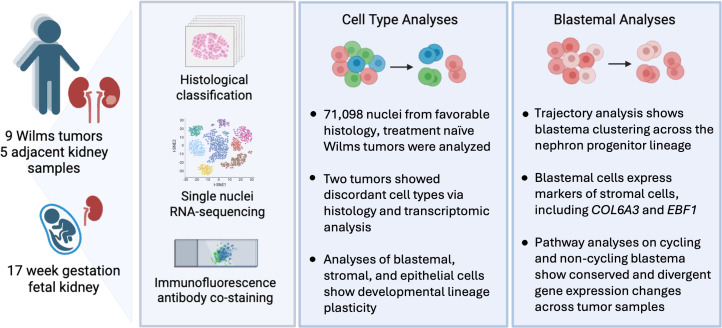

## Introduction

Wilms tumor, also known as nephroblastoma, is the most common kidney cancer in children (1). While most patients have an overall good prognosis, current treatment regimens are associated with long-term, adverse effects in survivors, and those patients with unfavorable histology and/or recurrent disease have worse outcomes (2, 3). Two treatment guidelines have been established by international multidisciplinary cooperative consortia, with the International Society of Paediatric Oncology (SIOP) regimens relying on preoperative chemotherapy followed by surgery and postoperative chemotherapy, versus the Children’s Oncology Group (COG) Renal Tumour Committee trials utilizing primary surgical resection prior to adjuvant therapies for the approximately 80% of unilateral Wilms tumors when such upfront resection is deemed feasible (4). Given the need to develop more effective and targeted therapies, further understanding of the complex, heterogeneous landscape of this embryonal tumor has the potential to advance biology-driven approaches and novel drug discovery in the treatment of Wilms tumor (4–6).

Wilms tumors arise from renal precursor cells and maintain features of the embryonic kidney, consisting of blastemal, epithelial, and stromal (also referred to as “interstitial”) cells. While the link between Wilms tumorigenesis and the dysregulation of normal development has long been recognized and is further detailed in a number of recent reviews (7–10), the mechanisms underlying the malignant transformation of renal precursor cells and cell type(s) of origin remain incompletely understood. Given this, there remain substantial opportunities to further leverage an understanding of Wilms tumor biology to advance clinical diagnostics, risk stratification, and the development of novel therapeutics. Single-cell transcriptomics has proven to be a powerful tool in the study of development and disease, as it overcomes the limitations of traditional RNA sequencing methods by measuring the whole transcriptome at single-cell resolution, thus distinguishing different cell types in tumor tissue (11) and offering the potential to provide new insights into understanding the cell type(s) of origin and the transcriptional trajectories driving the malignant transformation of renal precursor cells. As such, single-cell techniques have been used to define transcriptional profiles of cell types in the developing kidney (12–14) as well as to investigate Wilms tumors biology (15–17), as summarized in a recent review (18). However, the initial studies pioneering this work utilized samples obtained after chemotherapy (15, 16, 19, 20), as per the SIOP treatment guidelines, with chemotherapy known to induce genetic and transcriptional changes when compared with treatment-naive specimens in a number of various cancers. Specifically in Wilms tumor, a study evaluating postchemotherapy versus treatment-naive samples identified differences in the transcriptional signatures, including loss of nephron progenitor cell (NPC) genes in the postchemotherapy samples, although this evaluation was limited by the use of only a single treatment-naive sample (15). Thus, further evaluation of treatment-naive tumors offers an important opportunity to additionally evaluate how Wilms tumors both maintain features and diverge from their developmental origins, given the robust single-cell sequencing data of the human fetal kidney generated in the study of normal development.

Single-cell technologies additionally offer insights into the cellular heterogeneity of biologically complex tissues. While the clinical variability and diverse histological elements of Wilms tumors have long been recognized, recent genome sequencing studies have uncovered surprising heterogeneity in Wilms tumors, as evidenced by over 40 different genetic drivers (6, 21, 22), with the most common causative mutations found in only 10%–15% of tumors and the majority of identified drivers each found in less than 5% of tumors (23). Further complicating the genetic landscape of Wilms tumor, studies investigating pediatric tumor phylogenetics have identified intratumor heterogeneity, various evolutionary trajectories, as well as subclonal changes related to chemotherapy (24, 25). Such studies have uncovered postzygotic founder mutations (e.g., somatic 11p15 loss of heterozygosity), which may reflect germline mosaicism that emerged prior to the divergence of left and right kidney primordia in patients with bilateral tumors, versus other genetic perturbations (e.g., 1q gain, for example) that appear more heterogeneously identified throughout tumors (24, 26) and may complicate the detection of biomarkers aimed at determining prognosis and risk stratification, thus highlighting the importance of evaluating intratumoral heterogeneity.

Given the gaps described above, we sought to further investigate the cellular and molecular heterogeneity of Wilms tumors by correlating single-nucleus RNA sequencing (snRNA-seq) with tumor histology to further map the cell-type-specific landscape of treatment-naive, favorable-histology Wilms tumors. By specifically evaluating how these embryonal tumors both recapitulate and diverge from normal human fetal kidney tissue, we identify gene expression changes in tumor cells consistent with developmental-lineage plasticity, suggesting that transcriptional profiling may further inform the histological classification of some tumors. Additionally, despite the homogeneous appearance of the blastemal component at the histological level, we show heterogeneity in the expression of a number of markers by immunofluorescence. Furthermore, preliminary insights into potential molecular targets of the blastema, a critical source for chemoresistant clones, are also explored and identify a number of key signaling pathways, highlighting both conserved and divergent features of the 9 sequenced tumor samples in this study.

## Results

### Single-nucleus profiling of favorable-histology Wilms tumor integrated with a reference atlas including adjacent kidney tissue and human fetal kidney identifies distinct tumor cell types.

snRNA-seq was performed on favorable-histology Wilms tumor samples obtained from surgical resection specimens prior to chemotherapy in addition to histologically normal-appearing adjacent kidney tissue samples biobanked at the time of surgery. Samples in this study included 9 tumors, 4 patient-matched samples from corresponding tumors, and 1 additional adjacent kidney sample (Table 1), as this provided samples from both male/female adjacent kidney tissue to be included in the reference atlas.

**Figure 1:**
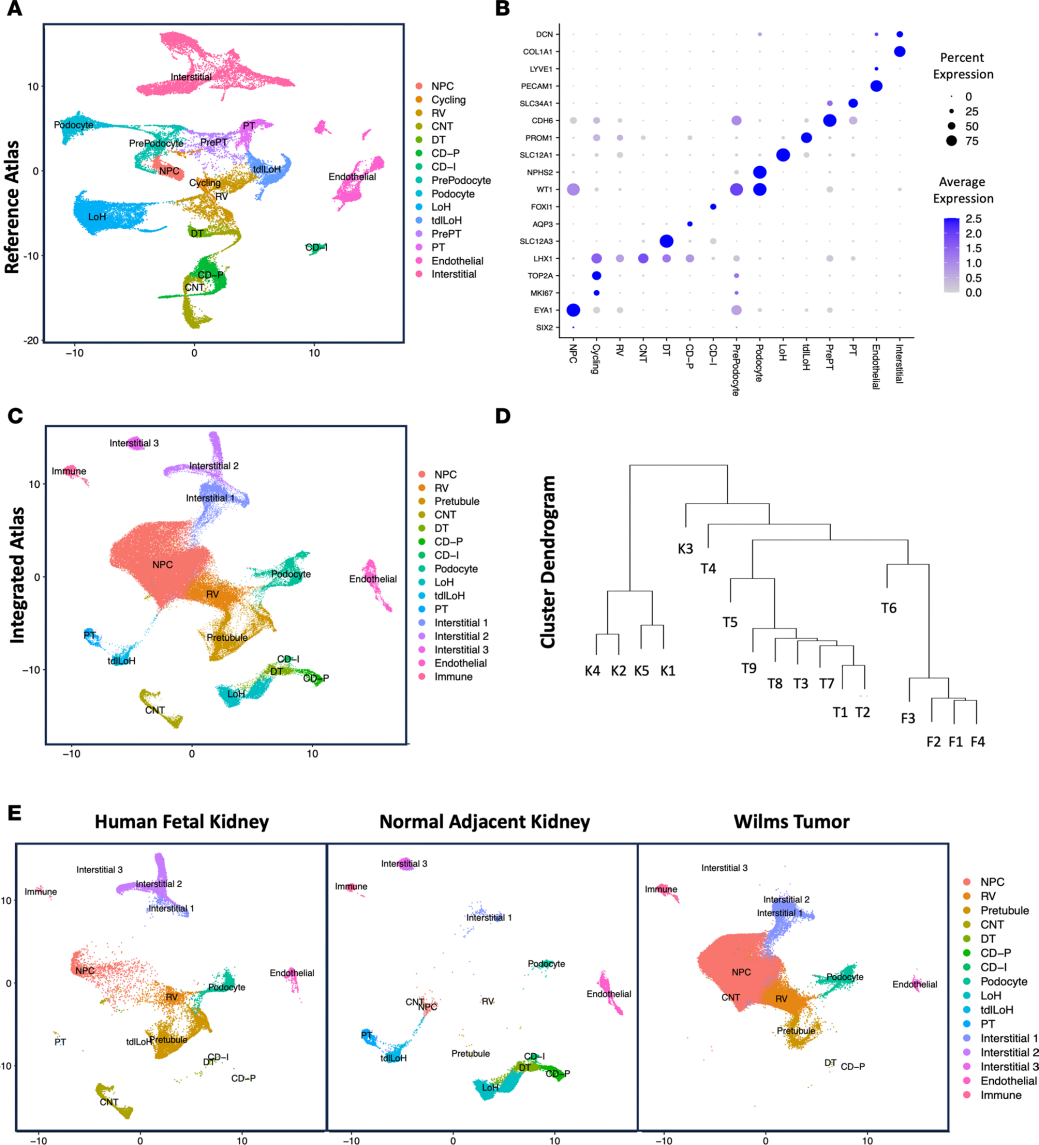
snRNA-seq identifies diverse cell types from favorable-histology Wilms tumor samples. (**A** and **B**) To evaluate how tumors maintain features of their developmental origins, we generated an snRNA-seq reference atlas consisting of 4 samples from 17-week-gestation human fetal kidney along with 5 tumor-adjacent kidney samples confirmed to be histologically normal kidney as reviewed by a pathologist prior to sequencing (**A**). Marker gene expression was used to identify captured cell types, with representative genes shown via dot plot (**B**). (**C**–**E**) To identify the cell types captured from Wilms tumor samples, snRNA-seq from 9 tumors was integrated with the reference atlas (**C**). Hierarchical analysis (i.e., cluster dendogram) shows transcriptional similarity of Wilms tumors with human fetal kidney in comparison to tumor-adjacent kidney samples (**D**). Tumor samples consist of a substantial proportion of blastemal cells clustering with human fetal nephron progenitors, early differentiating nephron structures (i.e., renal vesicles), and early tubule cells (i.e., pretubule), in addition to capturing other cell types including interstitial, endothelial, and immune cells (**E**). NPC, nephron progenitor cell; RV, renal vesicle; CNT, connecting tubule; DT, distal tubule; CD-P, collecting duct principal cell; CD-I, collecting duct intercalated cell; LoH, loop of Henle; tdlLOH, thick descending limb of loop of Henle; PrePT, precursor proximal tubule; PT, proximal tubule.

Given that Wilms tumors maintain features of the developing kidney, we first established a normal kidney reference (Figure 1A) from the 5 adjacent kidney samples (15,997 nuclei) along with human fetal kidney snRNA-seq (12), including 4 replicates of 17-week-gestation fetus (19,330 nuclei) that were integrated into a single data set, resulting in a total of 35,327 nuclei after preliminary filtering. Expected cell types are identified in the data set (Figure 1A), with representative marker genes shown via dot plot for cell clusters (Figure 1B), including NPCs (*SIX2, EYA1*), cycling NPCs (*TOP2A*, *MKI67*), early differentiating cells/renal vesicles (RVs) (*LXH1*), early proximal tubules (*CDH6*), proximal tubules (*SLC34A1*), podocytes (*NPHS2*), loop of Henle (*SLC12A1*), distal tubules (*SLC12A3*), collecting duct principal (*AQP3*) and intercalated (*FOXI1*) cells, endothelial cells (*PECAM1*), and interstitial (e.g., stromal) cells (*COL1A1*, *DCN*). As shown in Figure 1A, this provided a robust dataset from progenitor populations through the spectrum of differentiation to mature cell types, with a complete marker list for each annotated cell type provided in Supplemental Data File 1; supplemental material available online with this article; https://doi.org/10.1172/jci.insight.188078DS1

Next, snRNA-seq from the 9 Wilms tumor samples (totaling 71,098 nuclei) were integrated with the reference kidney atlas (Figure 1C). Hierarchical clustering analysis, with bulk transcriptomic data from each individual sample compared to one another and visualized via cluster dendrogram, suggests that tumors overall show a closer relationship to human fetal kidney compared with the adjacent kidney samples (Figure 1D). Tumor cell type annotation, including the blastemal, epithelial, or stromal (e.g., interstitial) compartments, was determined based on cell clustering in comparison with the reference atlas. This analysis identified tumor samples consisting of nephron lineage cells, including blastema clustering with fetal NPCs, early differentiating cells/RVs, and stroma (e.g., interstitial cells) from the fetal kidney samples, while podocytes, immune cells, and endothelial cells captured from tumors clustered with corresponding cell types from the adjacent normal kidney samples (Figure 1E). A full marker list of all the cell types is shown in Supplemental Data File 2.

**Figure 2:**
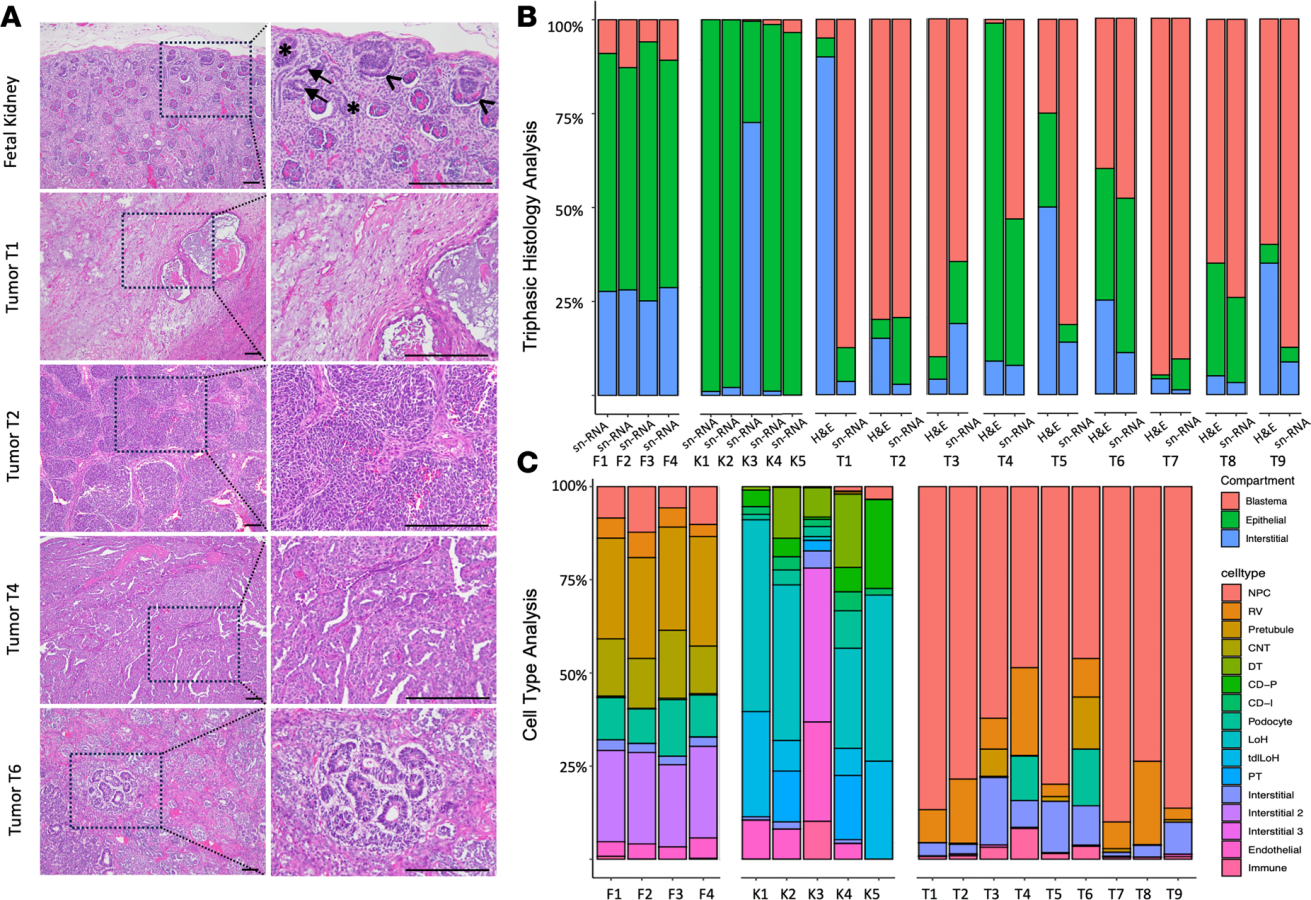
Histological examination of tumor samples correlates with cell types captured via snRNA-seq, with the exception of tumors T1 and T4. (**A**) H&E images of the fetal kidney compared to Wilms tumor samples show similar histological features. In the human fetal kidney, nephron progenitor cells (asterisks), early differentiating nephron structures (arrowheads), and early tubules (arrows) localize to the outer nephrogenic zone of the developing kidney, with Wilms tumors known to maintain features of these fetal cells. Representative H&E images of human fetal kidney and selected tumors are shown here. Original magnification, ×10 (left) and ×20 (right). Scale bars: 100 μm. (**B**) Tumor histology was scored by a pathologist to quantify the percentage of blastemal, epithelial, and stromal compartments from each sample. While the majority of tumors showed correlation between the tumor components captured via snRNA-seq and the histological score, there were discrepancies noted for T1, which showed interstitial (i.e., stromal) predominance by histology versus blastemal predominance by snRNA-seq and for T4, which showed epithelial predominance by histology versus blastemal predominance by snRNA-seq. (**C**) Evaluation of the specific cell types captured (i.e., percentage cell type) is shown for each sample, demonstrating populations of cells along the spectrum of the nephron lineage, including undifferentiated NPCs to RVs/pretubules, found to within tumor samples. NPC, nephron progenitor cell; RV, renal vesicle; CNT, connecting tubule; DT, distal tubule; CD-P, collecting duct principal cell; CD-I, collecting duct intercalated cell; LoH, loop of Henle; tdlLOH, thick descending limb of loop of Henle; PT, proximal tubule.

To evaluate how the captured cell types by snRNA-seq correlate with the histological classification of the tumor samples, H&E-stained tissues adjacent to the sample used for snRNA-seq were reviewed by a pathologist to quantify the percent of blastemal, epithelial, and stromal compartments from each sample, with representative images from selected tumors shown in comparison to human fetal kidney (Figure 2A). While the snRNA-seq data overall correlate well with the histological elements on H&E (Figure 2B and Supplemental Tables 1 and 2), we notably identified 2 tumors showing discordant findings. This includes sample T1, which shows stromal predominance by histology but primary blastema captured by snRNA-seq, and T4, which shows epithelial predominance by histology but with an increased percentage of blastema captured by snRNA-seq. Further evaluation of specific cell types captured (Figure 2C) shows tumor cells clustering with various cell types along the nephron lineage (i.e., NPCs, RVs, pretubule cells, and podocytes). Adjacent kidney samples show epithelial predominance, with the exception of sample K3 that shows an increased proportion of interstitial and endothelial cells captured by snRNA-seq possibly due to sampling of a vascular or medullary region of the kidney. Additionally, a small percentage of blastemal cells were captured in the adjacent, histologically normal samples, including 3% in K5 and 1.2% in K4. It is unclear whether these cells could be due to tumor margins close to the sampled tissue, as has been reported previously (27), versus blastemal cells possibly infiltrating the normal kidney tissue or contamination during surgical resection, but given this small number of cells, these were not included in further analyses.

### Transcriptional profiles from blastemal, stromal, and epithelial components shows multilineage plasticity.

**Figure 3:**
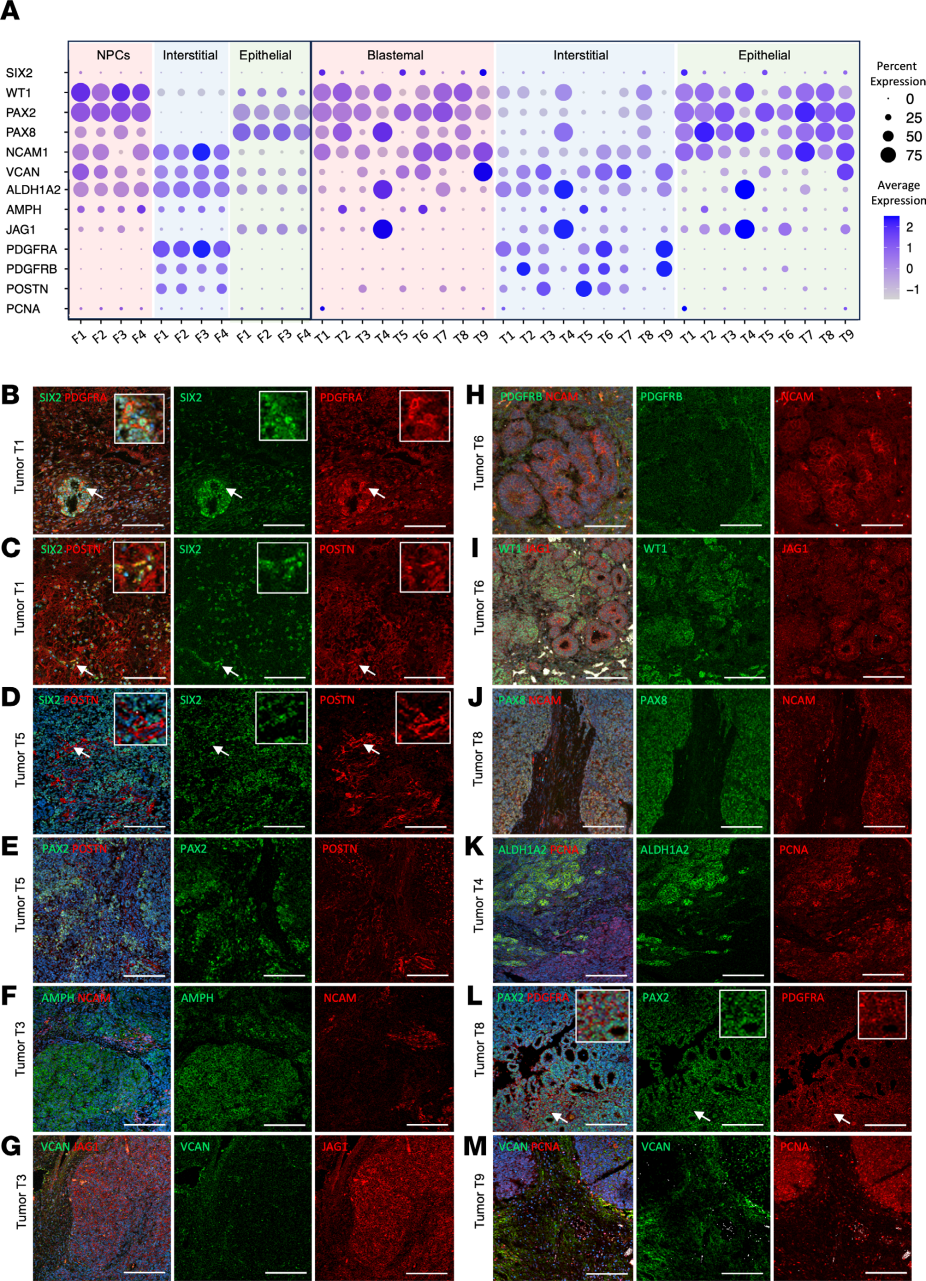
Blastemal, stromal, and epithelial components from favorable-histology Wilms tumor show multilineage plasticity. (**A**) The expression pattern of selected genes from the snRNA-seq dataset was evaluated across the NPC/blastema, stromal, and epithelial components of human fetal kidney and tumor snRNA-seq data. In the fetal kidney, several genes show cell-type-specific expression patterns, with *SIX2* and *WT1* primarily expressed in NPCs, *PAX2* and *PAX8* expressed in the nephron lineage (i.e., NPCs and differentiating epithelial cells, but not the interstium/stroma), and *PDGFRA*, *PDGFRB*, and *POSTN* expressed in the interstitium. Tumor snRNA-seq shows misexpression of several lineage-specific genes, including *SIX2* expression in epithelial cells of T1 and T5, *POSTN* expression in the blastema of T3, T5, and T7, and *PAX8* expression in the interstitium of T4 with both *PAX2* and *PAX8* in T8. (**B–M**) Representative images of immunofluorescence validation confirms expression of the selected markers in tumor samples, with costaining showing misexpression of a number of lineage-specific markers. Specifically, SIX2-expressing cells appear to show coexpression of PDGFRα (**B**, arrows) and POSTN (**C** and **D**, arrows). Additionally, PAX2 appears coexpressed with PDGFRα in some regions of T8 (**L**, arrow). Representative images of tumors from at least 2 separately stained sections are shown at ×10 magnification. Scale bars: 100 μm.

While previous single-cell transcriptomic studies have shown that Wilms tumors maintain fetal transcriptional profiles (15, 16), these studies were limited by the use of samples obtained after chemotherapy. Here, treatment-naive samples were sequenced and selected genes showing specific expression patterns in human fetal kidney cell types were evaluated in tumor components (Figure 3A). In the developing kidney, several genes show cell-type-specific expression patterns, with *SIX2* and *WT1* expressed in NPCs, *PAX2* and *PAX8* expressed in the nephron lineage (i.e., NPCs and differentiating epithelial cells) but not the interstium/stroma, and several genes (i.e., *PDGFRA*, *PDGFRB*, and *POSTN*) showing expression in the interstitium but not the nephron lineage. Analysis of these selected genes in tumor components shows misexpression of several lineage-specific markers, including *SIX2* expression in epithelial cells of T1 and T5, *POSTN* expression in the blastema of T3, T5, T7, and T8, and *PAX2* and *PAX8* expression in the interstitium of T4 and T8. Immunoassay validation for the selected markers was performed (Figure 3B), with coexpression of the NPC marker SIX2 and stroma-specific gene PDGFRα observed in specific cells of sample T1. Additionally, stromal cells marked by POSTN appear to show nuclear SIX2 expression in T1 and T5, which may account for the discrepancy in which the histology of sample T1 showed stromal predominance by H&E but the snRNA-seq data showed cell types clustering with the blastema. Furthermore, immunoassay validation showed cellular heterogeneity for a number of markers, including SIX2 and PAX2 expression in T5 as well as WT1 in T6, ALDH1A2 in T4, VCAN in T9, and the proliferation marker PCNA in T4 and T9.

### Wilms tumor blastema shows both intra- and intertumor heterogeneity.

**Figure 4:**
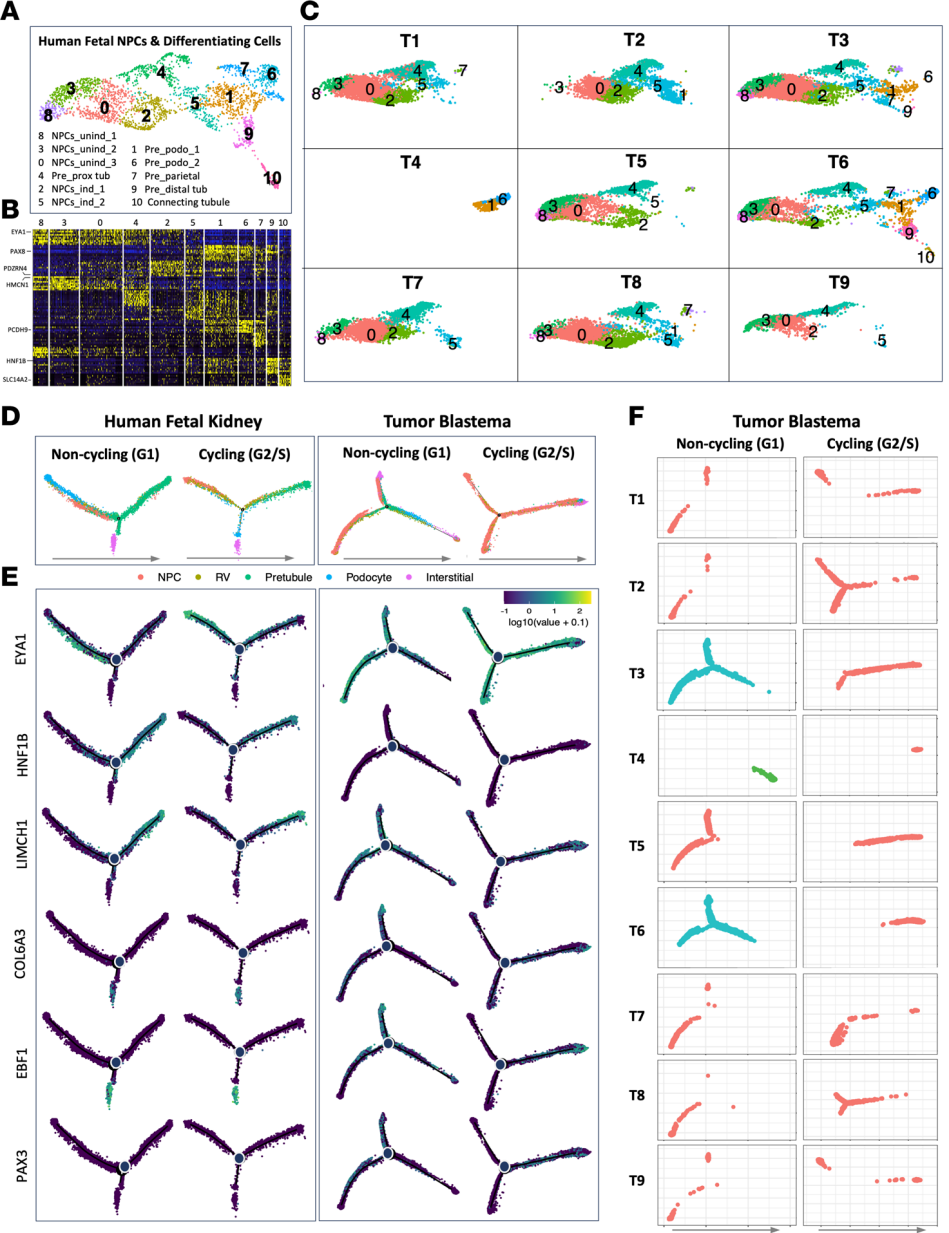
Trajectory analysis of Wilms tumor blastema shows intra- and intertumor heterogeneity. (**A** and **B**) To evaluate how tumor blastema resembles the differentiation trajectory of the nephron lineage, we further analyzed human fetal kidney snRNA-seq samples to identify subclusters of nephron lineage along the differentiation trajectory from self-renewing NPCs to differentiated cells. Unsupervised cell clustering identified 10 clusters (**A**) based on differential transcriptional profiles with selected genes highlighted in this heatmap (**B**), with this analyses identifying 3 subclusters of uninduced NPCs (i.e., 8, 3, and 0), 2 subclusters of induced NPCs (i.e., 2 and 5), and precursor populations for early podocytes (i.e., 1 and 6), proximal tubules (i.e., 4), parietal cells (i.e., 7), distal tubules (i.e., 9), and connecting tubules (i.e., 10). (**C**) Integration of tumor blastema from each of the 9 samples shows that the blastema from the majority of tumors clusters with cells along the spectrum of normal fetal kidney differentiation trajectory, with the exception of T4 that clusters exclusively podocyte precursors. (**D**–**F**) To further evaluate this, we first pooled the blastema from all 9 samples and analyzed the differentiation trajectory of noncycling versus cycling cells. As a control, fetal kidney cells were analyzed via the same methodology, with interstitial cells clustering separately from the nephron lineage. However, tumor blastema shows a mix of differential trajectories, with expression of pretubule, podocyte, and interstitial genes among the differentiation trajectories (**D**). Additionally, the cycling cells show enriched expression of NPCs genes (i.e., *EYA1*) and decreased expression of tubular markers (i.e., *HNF1B* and *LIMCH1*) along with both the cycling and noncycling tumor blastema showing misexpression of interstitial genes (i.e., *COL6A3* and *EBF1*) and *PAX3*, which is normally not expressed in the human fetal kidney (**E**). Differential trajectories were then evaluated for the noncycling and cycling blastema from each individual tumor (**F**). Color coding shows representation along the differentiation trajectory, with orange representing NPC signature, green representing an epithelial signature, and blue representing a mixed signature.

We next sought to further evaluate how the blastemal populations isolated from 9 favorable-histology Wilms tumors compare to fetal kidney NPCs and the developing nephron lineage. To do this, we first reevaluated human fetal kidney NPCs and early differentiating cells for subclusters showing distinct transcriptional profiles (Figure 4, A and B, with differentially expressed genes [DEGs] distinguishing the subclusters provided in Supplemental Data File 3). This analysis identified a total of 10 subclusters, including 3 subclusters of self-renewing or “uninduced” NPCs, 2 sublclusters of “induced” NPCs, as well as precursor cells of the proximal tubules, podocytes, parietal cells, distal tubule, and connecting tubule. Blastemal cells were then integrated with the human fetal kidney cell clusters (Figure 4C), with this analysis suggesting that the blastema from the majority of tumors clusters with cells along the spectrum of normal fetal kidney differentiation trajectory, with the exception of sample T4 that clusters exclusively with podocyte/podocyte precursor cells.

Given the proliferative capacity that has been previously reported in Wilms tumor blastema, we next analyzed the differentiation trajectory of the noncycling versus cycling blastema, with human fetal kidney cells analyzed via the same methodology for comparison. Monocle was used to order the isolated blastemal cells based on their transcription factor expression profile to determine the differentiation state of the cells. Given the lineage plasticity previously identified (i.e., Figure 3) with blastemal cells showing expression of genes normally localized to interstitial/stromal cells, we additionally included stromal cells of the human fetal kidney in the control analysis as a comparison when mapping expression of stromal marker genes as well. As expected, both cycling and noncycling human fetal kidney NPCs cluster along a differentiation trajectory from uninduced NPCs to tubular precursor cells plotted left to right, with the stromal/interstitial cells plotting as a separate cluster. However, this analysis of tumor blastema interestingly shows a loss of this ordered differentiation trajectory (Figure 4, D and E), with the cycling cells showing increased expression of the uninduced NPC marker *EYA1*, a lack of the differentiating NPC marker *HNF1B*, and misexpression of the distal tubular marker *LIMCH1* and stromal genes *COL6A3* and *EBF1* as well as *PAX3*. Interestingly, although cycling cells from the tumor blastema show a more undifferentiated signature, reflected by elevated *EYA1* expression, they continue to express stromal genes (e.g., *COL6A3* and *EBF1*) and differentiation-associated markers (e.g., *LIMCH1*). When evaluating the differentiation trajectory of individual tumors (Figure 4F), the patterns of “more” or “less” differentiated tumors emerge, with T1, T2, T5, T7, T8, and T9 showing a more “undifferentiated” signature, while T3, T4, and T6 blastema included cells with a more “differentiated” signature based on this trajectory analysis, with orange representing an NPC signature, green representing an epithelial signature, and blue representing a mixed signature.

### Pathway enrichment analyses provide insights into both cycling and noncycling blastema in Wilms tumor.

**Figure 5:**
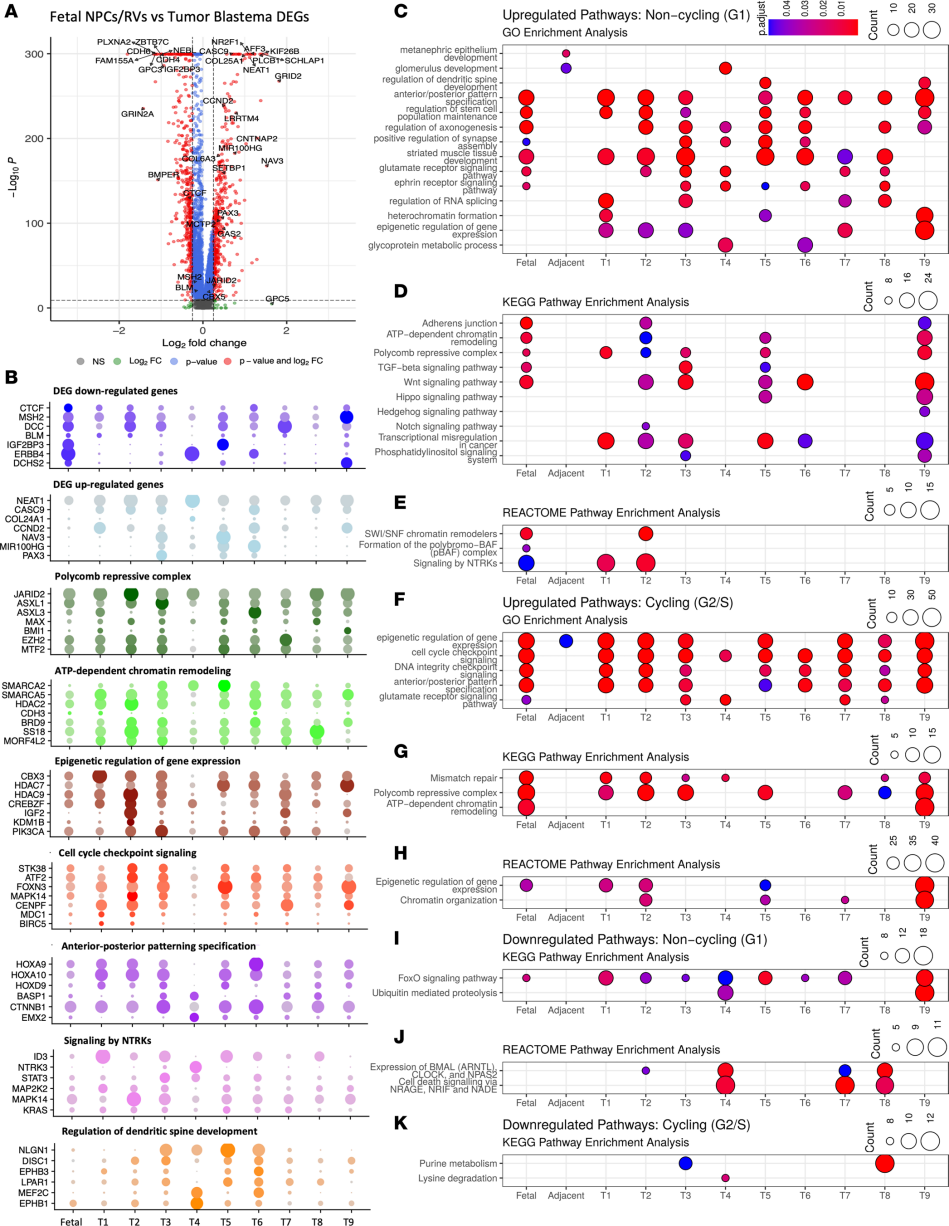
Differentially expressed gene (DEG) analysis of Wilms tumor blastema further demonstrates both intra- and intertumor heterogeneity. (**A** and **B**) To evaluate for transcriptional changes in tumor blastema versus human fetal kidney NPCs and early differentiating cells, we first pooled cells these cells from all the samples in the 2 groups and analyzed for DEGs as shown via volcano plot (**A**). Given the heterogeneity observed across tumor samples, we next evaluated selected downregulated and upregulated gene expression across the 9 tumors via dot plots (**B**) with selected genes from the DEG analyses shown above as well as the pathway enrichment analysis highlighted via dot plot. (**C**–**K**) Pathway analysis was performed separately both the noncycling and cycling blastema, with this analysis highlighting a number of transcriptional pathways and demonstrating remarkable intertumor heterogeneity in the blastemal cells from favorable-histology Wilms tumors. Notably, the analyses of cycling cells from adjacent kidney samples showed insufficient genes for KEGG and Reactome (**G** and **H**, respectively) and were not included in these analyses.

To further evaluate how the transcriptome from tumor blastema compares to fetal kidney NPCs/RVs, we identified DEGs and evaluated these transcriptomic changes in the context of biological pathway analyses. First, blastemal cells from the 9 tumor samples were pooled and compared to fetal kidney NPCs/RVs, with DEGs shown via volcano plot (Figure 5A and Supplemental Data File 4). To evaluate for intertumor transcriptional heterogeneity, we next examined the expression of top DEGs across the 9 tumor samples (Figure 5B) and found that some gene expression changes were fairly uniformly conserved (e.g., downregulation of *CTCF* and upregulation of *NEAT1* in comparison with fetal kidney cells), whereas a number of other genes showed striking differences among the individual tumor samples (e.g., downregulation of *MSH2* and *ERBB4* and upregulation of *CCND2* and *PAX3*). Given this heterogeneity in gene expression, we sought to perform pathway enrichment analyses to potentially uncover both common and distinct pathways in the tumor blastema that may be difficult to predict from individual gene expression changes alone. To do this, Gene Ontology (GO) biological processes, Kyoto Encyclopedia of Genes and Genomes (KEGG), and Reactome pathway enrichment analyses were performed separately on the noncycling and cycling blastema cells compared to normal adjacent kidney tissue as well as fetal NPCs/RVs (Supplemental Data File 5), with selected pathways shown in Figure 5, C–K. As expected, gene pathways for renal epithelium and glomerulus development were enriched in the normal adjacent kidney tissue compared with the fetal cells and tumor blastema (Figure 5C), with the exception of T4 that also showed enrichment for glomerulus development consistent with podocyte signature identified in the previous clustering analysis (Figure 4C). We additionally identified a number of enriched pathways across several tumor samples, including regulation of stem cell population maintenance, regulation of axonogenesis, striated muscle development, glutamate receptor signaling, and ephrin receptor signaling, all of which show shared features with human fetal kidney cells. However, additional identified pathways, including regulation of RNA splicing, heterochromatin formation, epigenetic regulation of gene expression, and transcriptional misregulation in cancer appear enriched in certain samples of tumor blastema. When looking at pathways enriched in the cycling blastemal cells, we identify cell cycle checkpoint signaling, DNA integrity checkpoint signaling, Polycomb repressive complex (PRC), and ATP-dependent chromatin remodeling. We also compared genes that were downregulated across the samples, with this analysis suggesting decreased FoxO signaling, ubiquitin mediated proteolysis, circadian rhythm regulation via BMAL, CLOCK, and NPAS2, as well as cell death signaling. Given the heterogeneity observed across tumor samples, we additionally evaluated selected genes from specific pathways (Figure 5B), including PRC, ATP-dependent chromatin remodeling, epigenetic regulation, cell cycle checkpoint signaling, anterior-posterior patterning specification, signaling by neurotrophic receptor tyrosine kinases (NTRKs), and regulation of dendritic spine development. This analysis interestingly confirms gene expression changes corresponding to identified pathways and further demonstrates that such types of analyses may aid in the approach to understanding transcriptional heterogeneity in Wilms tumor.

## Discussion

In this study, the use of a comprehensive reference atlas, including normal adjacent kidney tissue and 17-week human fetal kidney, was leveraged to facilitate the analysis of cell-type-specific transcriptional profiles from favorable-histology, treatment-naive Wilms tumor. While Wilms tumors are known to demonstrate triphasic histology, here we identify transcriptional plasticity within the tumor cell types, as demonstrated by markers of the nephron lineage expressed in tumor stroma, as well as genes normally restricted to the renal interstitium expressed in the tumor blastema. To our knowledge, this report is the first to demonstrate such aberrant gene expression changes suggesting transcriptional reprogramming and developmental-lineage plasticity at the single-cell level. Specifically, the blastemal cells from each of the 9 tumors sampled in this study show transcriptional profiles consistent with the nephron lineage, including “uninduced” and “induced” NPCs that differentiate to form early epithelial structures including podocytes, consistent with findings from previous studies (28, 29). Interestingly, 8 of the 9 tumor samples showed blastemal cells that shared transcriptional profiles with subclusters of the normal fetal NPCs, while 1 tumor (e.g., T4) clustered with podocyte precursor cells (Figure 4C). Furthermore, trajectory analyses suggest that while the cycling blastema show aberrant gene expression, as evidenced by stromal markers and *PAX3* expression, these cells additionally show enriched expression of “uninduced” NPC markers (Figure 4, D–F). Overall, these findings uncover important features regarding the ontogeny of the blastema and suggest that transcriptional profiles may provide further insights beyond histological classification. Whether the “developmental lineage plasticity” identified here arises from defects in early lineage specification or from the ability of tumor cells to “dedifferentiate” to more primitive precursors cells, and how the loss of normal lineage ontogeny may contribute to the malignant transformation of NPCs will require additional studies.

Given the aberrant gene expression identified in Wilms tumor, pathway enrichment analyses offer the potential to reveal biologically relevant signaling in tumorigenesis. Pathway analytics confirmed WNT/β-catenin signaling in tumor blastema (Figure 5D). WNT/β-catenin has been, until recently, an evasive oncogenic target in many cancers, including embryonic tumors such as Wilms tumor (30). More recently, tegavivint, targeting β-catenin, has advanced to phase I/II investigation in pediatrics, including Wilms tumor (i.e., ClinicalTrials.gov NCT04851119). While correlative analytics have largely focused on β-catenin–activating mutations, data herein suggest consideration for subcellular profiling potentially being an additional predictive biomarker of potential efficacy. Pathway analyses also identify the PRC enriched in the blastema of 5 of the 9 tumors sequenced (Figure 5, B and D). Targeting cancers with perturbed PRC function has also advanced, in particular for the treatment of tumors harboring SMARCB1 mutations such as kidney rhabdoid tumors and other cancers (31). EZH2 inhibition in particular, functioning as a subunit of the PRC, has demonstrated both in vitro and in vivo anticancer activity (32, 33). As suggested by our analysis here, whether subsets of Wilms tumors will be vulnerable to clinical targeting of the PRC via EZH2 inhibition or alternative epigenetic modulation and/or chromatin remodeling remains to be defined.

Additional pathways highlighted in this analysis include cell cycle checkpoint signaling, anterior-posterior patterning (showing misregulation of the *HOX* gene family), signaling by NTRKs, and regulation of dendritic spine (e.g., synaptic protrusions of neurons), which we examined closer by comparing specific gene expression from each pathway across the individual tumor samples in comparison to fetal NPCs (Figure 5B). This analysis identified interesting heterogeneity not only across tumors, but within specific tumors as well. For instance, while cell cycle checkpoint signaling is overall upregulated in the blastema of the majority of tumors sampled, T4 interestingly shows a lack of signal even in comparison to fetal NPCs, and individual tumors show differing patterns in the expression of pathway-specific genes. A similar pattern emerges for NTRK signaling, with specific tumors enriched with various regulatory genes from this pathway, which may be of interest given the known role of fusion protein in various cancers and the development of TRK inhibitors (34). Other pathways of interest identified include upregulation of Hippo, Hedgehog, and Notch signaling, loss of adherens/tight junctions, and downregulation of FoxO signaling, which has been shown to regulate a number of cellular processes including cell cycle arrest (35). Global suppression of cell death signaling in Wilms tumor may suggest the potential for therapeutic modulation of the apoptotic program (36).

While this study includes 9 favorable-histology, treatment-naive Wilms tumor samples, cohorts with increased tumor samples will likely be necessary to fully characterize intertumor heterogeneity uncovered in this study. Additionally, further studies defining the spatial relationship of cycling/noncycling cells, blastemal populations, as well as cells demonstrating lineage plasticity are likely to provide additional insights into the multiphasic nature of these embryonal tumors. Despite these limitations, the clinical applications of single-nucleus RNA profiling in Wilms tumor are numerous, including, but not limited to, potential diagnostic applications, advancing biologic understandings related to Wilms tumor oncogenesis from the nephrogenic rest stage through anaplasia and advanced metastases, identification of biology and targets that address chemotherapy resistant clones, and uncovering cellular pathways amenable to targeted therapy in Wilms tumor subsets. While a number of these applications are outside of the scope of this study, our findings from this analysis of the transcriptional landscape of treatment-naive Wilms tumor further suggest that enabling precision medicine approaches may lead to advances in individualizing approaches to children with Wilms tumor.

## Methods

### Sex as a biological variable.

This study included both male and female clinical samples. Sex was not considered as a biological variable due to the lack of statistical power in this study including 9 Wilms tumor samples.

### Clinical specimens, sample preparation, histology, and immunofluorescence.

Wilms tumor samples were obtained from patients undergoing clinical nephrectomy/surgical resection at Cincinnati Children’s Hospital Medical Center. With appropriate consent, representative samples of tumor tissue and, when available, matched grossly normal renal parenchyma were collected at the time of nephrectomy. Specimens were either snap frozen and stored at −80°C in the Cincinnati Children’s institutional tissue biobank or preserved as formalin-fixed paraffin-embedded (FFPE) tissue. For this study, the biobank records were reviewed and 9 Wilms tumor samples, 4 of which had paired background renal parenchyma available, were requested for snRNA-seq, and during the study period, 1 active case was able to be sampled in real time. These were selected to include a total of 9 previously untreated Wilms tumors of favorable histology. The frozen storage time for the tissue samples ranged from 45 minutes for the active case to 25 years in the biobank.

Where circumstances permitted, H&E staining was performed on the frozen tissue section before sequencing and was used for histologic analysis. If this could not be done, a permanent H&E section from an FFPE block adjacent to the sample was used. H&E sections were reviewed by a pathologist for confirmation of the presence or absence of anaplastic features. In addition, percentages of blastemal, epithelial, and stromal elements were estimated and subsequently compared to the cell populations identified by sequencing. Blastemal elements were defined as areas showing sheets and nests of undifferentiated tumor cells with a high nucleus/cytoplasm ratio, with epithelial elements defined as primitive tubular or glomeruloid structures within the tumor. Stromal elements were predominantly areas of bland, spindled mesenchyme, although any heterologous mesenchymal element (including rhabdomyomatous differentiation) was also included in this category.

Immunofluorescent staining was performed on FFPE tissue sectioned to 5 μm slices. Slides for immunofluorescence were immersed and boiled with either 10 mM sodium citrate or TE antigen retrieval buffer and blocked with a solution of 5% normal donkey serum for 1 hour at room temperature followed by the application of primary antibodies diluted in blocking solution. The following primary antibodies were used: SIX2 (Proteintech, 11562-1-AP; 1:200 dilution; rabbit; RRID: AB_2189084), PDGFRα (R&D Systems, AF1062; 1:200 dilution; goat, RRID: AB_2236897), POSTN (Santa Cruz Biotechnology, sc-398631; 1:100 dilution; mouse; RRID: AB_2166653), PAX2 (BioLegend, 901001; 1:200 dilution; rabbit; RRID: AB_291611), AMPH (Proteintech, 13379-1-AP; 1:200 dilution; rabbit; RRID: AB_2226789), NCAM (Sigma-Aldrich, C9672; 1:200 dilution; mouse; AB_1079450), VCAN (Novus, NBP1-85432; 1:200 dilution; rabbit; RRID: AB_11006006), JAG1 (Novus, AF599-SP; 1:200 dilution; goat; RRID: AB_2128257), PDGFRB (Cell Signaling Technology, 3169S; 1:200 dilution; RRID: AB_2162497), WT1 (Abcam, ab89901; 1:200 dilution; rabbit; RRID: AB_2043201), PAX8 (Proteintech, 10336-1-AP; 1:200 dilution; rabbit; RRID: AB_2236705), ALDH1A2 (Sigma-Aldrich, HPA010022; 1:200 dilution; rabbit; RRID: AB_1844723), and PCNA (Abcam, ab18197; 1:200 dilution; rabbit; RRID: AB_444313). Imaging was performed on a NikonA1 inverted confocal microscope.

### snRNA-seq.

snRNA-seq was performed as previously described (37). Samples were sequenced on the Illumina NovaSeq 6000 platform. Resulting fastq files were processed through the CellRanger pipeline v6.1.2 (38) using 10X Genomics’ GRCh38-2020-A reference genome, with the setting “include introns” set to True.

Background reads were removed using decontX from the celda package using the filtered barcodes as the cells to keep and the remaining cells in the raw barcode matrix as the background (39). Doublets were minimized using DoubletFinder (40). The R v4.1.1 (R, 2021) library Seurat v4.9 (41) was used for cell type clustering and marker gene identification. Cells expressing more than 2000 genes were retained for downstream analysis. A normal kidney reference was generated by using the samples obtained from the histologically normal looking tissue and a human fetal kidney. Each sample was normalized SCTransform using the glmGamPoi method and the number of RNA molecules per cell was regressed out. Samples were integrated with common anchor genes using the rPCA method to minimize sample-to-sample variation. Cell clusters were determined by the Louvain algorithm using a resolution of 0.2. UMAP dimension reduction was done using the first 30 principal components. Marker genes for each cell type were calculated using the Wilcoxon rank-sum test returning only genes that are present in a minimum of 25% of the analyzed cluster. Monocle v2.2.4 (42, 43) was used to predict the developmental trajectory of the mesenchyme-like and RV-like cells of the Wilms’ tumor using the top 500 differentially expressed transcription factors to drive cell ordering. The R library clusterProfiler v4.12.2 was used to show pathway enrichments (44, 45).

### Statistics.

The R programming language was used for statistical analyses. scRNA-seq dimension reduction and clustering was performed through the Seurat v4.9 package. Dot plots were plotted via raw expression or scaled on the mean where appropriate. Cell-type DEGs were determined by Wilcoxon rank-sum test. Sample similarity was determined using the average expression of all genes from each sample, calculating their euclidean distances and plotting the hierarchical clustering with hclust. Pathways were determined significant if the *P* value was less than 0.05.

### Study approval.

The Institutional Review Board of Cincinnati Children’s approved this study (2019-0469).

### Data availability.

The snRNA-seq data generated in this study are available through the NCBI Gene Expression Omnibus under accession number GSE273662. The 17-week human fetal kidney snRNA-seq data are available under accession number GSE232479 (12). Values for all data points in graphs are reported in the Supporting Data Values file.

## Author contributions

JG, SSP, and MA conceptualized the study. MA, KAD, and KV developed the methodology and conducted the experiments. KV reviewed the H&E-stained tissue sections. JG acquired the funding. MA, KAD, NPS, and JG wrote the original manuscript draft. MA, KAD, NPS, KV, and JG participated in the editing and review of the manuscript. MA and KAD contributed equally, with MA performing critical bioinformatic analyses and KAD performing validation studies and critical revisions.

## Conflict of interest

The authors have declared that no conflict of interest exists.

## Funding support

This work is the result of NIH funding, in whole or in part, and is subject to the NIH Public Access Policy. Through acceptance of this federal funding, the NIH has been given a right to make the work publicly available in PubMed Central.

- NIH grant DK131258 (to KAD).
- UT Southwestern Simmons Comprehensive Cancer Center (SCCC) Pilot Program grant UTSW23001311 (to KAD).
- Cincinnati Children’s Hospital Medical Center Wilms Tumor Research Fund supported by the Sexton family and Wipe Out Wilms Tumor Foundation (to JG).

## Supplementary Material

Supplemental data

Supplemental data set 1

Supplemental data set 2

Supplemental data set 3

Supplemental data set 4

Supplemental data set 5

Supporting data values

## Figures and Tables

**Table 1:**
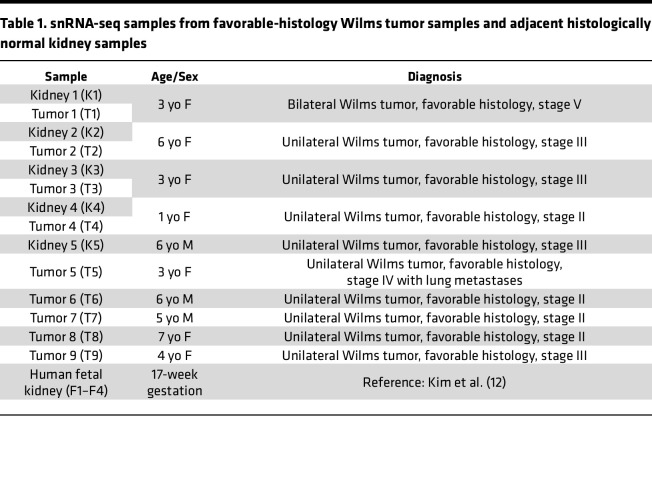
snRNA-seq samples from favorable-histology Wilms tumor samples and adjacent histologically normal kidney samples

## Version 1 06/09/2026

In-Press Preview

## Version 2 08/10/2026

Electronic publication

## References

[2] Huang CC (2009). Predicting relapse in favorable histology Wilms tumor using gene expression analysis: a report from the Renal Tumor Committee of the Children’s Oncology Group. Clin Cancer Res.

[3] Fawkner-Corbett DW (2014). Wilms’ tumor--lessons and outcomes--a 25-year single center UK experience. Pediatr Hematol Oncol.

[4] Spreafico F (2021). Wilms tumour. Nat Rev Dis Primers.

[5] Walz AL (2023). Tumor biology, biomarkers, and liquid biopsy in pediatric renal tumors. Pediatr Blood Cancer.

[6] Gadd S (2017). A Children’s Oncology Group and TARGET initiative exploring the genetic landscape of Wilms tumor. Nat Genet.

[7] Hastie ND (1994). The genetics of Wilms’ tumor--a case of disrupted development. Annu Rev Genet.

[8] Rivera MN, Haber DA (2005). Wilms’ tumour: connecting tumorigenesis and organ development in the kidney. Nat Rev Cancer.

[9] Hohenstein P (2015). The yin and yang of kidney development and Wilms’ tumors. Genes Dev.

[10] Li H (2021). Embryonic kidney development, stem cells and the origin of Wilms tumor. Genes (Basel).

[11] Zhang Y (2021). Single-cell RNA sequencing in cancer research. J Exp Clin Cancer Res.

[12] Kim S (2024). Comparative single-cell analyses identify shared and divergent features of human and mouse kidney development. Dev Cell.

[13] Lindström NO (2018). Conserved and divergent features of mesenchymal progenitor cell types within the cortical nephrogenic niche of the human and mouse kidney. J Am Soc Nephrol.

[14] Lindström NO (2021). Spatial transcriptional mapping of the human nephrogenic program. Dev Cell.

[15] Young MD (2021). Single cell derived mRNA signals across human kidney tumors. Nat Commun.

[16] Young MD (2018). Single-cell transcriptomes from human kidneys reveal the cellular identity of renal tumors. Science.

[17] Li T (2024). Single-cell transcriptomes of kidneys in a 6-month-old boy with Denys-Drash syndrome reveal stromal cell heterogeneity in the tumor microenvironment. Clin Kidney J.

[18] Pop NS (2025). Understanding developing kidneys and Wilms tumors one cell at a time. Curr Top Dev Biol.

[19] He TQ (2023). Development and validation of a prognostic model based on a single-cell RNA-seq in Wilms tumor in children. J Investig Med.

[20] Liu M (2024). Differentiation potential of hypodifferentiated subsets of nephrogenic rests and its relationship to prognosis in Wilms tumor. Fetal Pediatr Pathol.

[21] Mahamdallie S (2019). Identification of new Wilms tumour predisposition genes: an exome sequencing study. Lancet Child Adolesc Health.

[22] Treger TD (2025). Predisposition footprints in the somatic genome of Wilms tumors. Cancer Discov.

[23] Perotti D (2024). Hallmark discoveries in the biology of Wilms tumour. Nat Rev Urol.

[24] Cresswell GD (2016). Intra-tumor genetic heterogeneity in Wilms tumor: clonal evolution and clinical implications. EBioMedicine.

[25] de Sá Pereira BM (2019). Intra-tumor genetic heterogeneity in Wilms tumor samples. Rev Assoc Med Bras (1992).

[26] Coorens THH (2019). Embryonal precursors of Wilms tumor. Science.

[27] Darmanis S (2017). Single-cell RNA-Seq analysis of infiltrating neoplastic cells at the migrating front of human glioblastoma. Cell Rep.

[28] Stevenson MJ (2023). Altered binding affinity of SIX1-Q177R correlates with enhanced WNT5A and WNT pathway effector expression in Wilms tumor. Dis Model Mech.

[29] Trink Y (2023). Characterization of continuous transcriptional heterogeneity in high-risk blastemal-type Wilms’ tumors using unsupervised machine learning. Int J Mol Sci.

[30] Choudhary S (2024). Wnt/β-catenin signaling pathway in pediatric tumors: implications for diagnosis and treatment. Children (Basel).

[31] Gadd S (2010). Rhabdoid tumor: gene expression clues to pathogenesis and potential therapeutic targets. Lab Invest.

[32] Kim KH, Roberts CW (2016). Targeting EZH2 in cancer. Nat Med.

[33] Chi SN (2023). Tazemetostat for tumors harboring SMARCB1/SMARCA4 or EZH2 alterations: results from NCI-COG pediatric MATCH APEC1621C. J Natl Cancer Inst.

[34] Hechtman JF (2022). NTRK insights: best practices for pathologists. Mod Pathol.

[35] Hornsveld M (2018). Re-evaluating the role of FOXOs in cancer. Semin Cancer Biol.

[36] Takamizawa S (2000). Differential apoptosis gene expression in pediatric tumors of the kidney. J Pediatr Surg.

[37] May-Zhang AA (2021). Combinatorial transcriptional profiling of mouse and human enteric neurons identifies shared and disparate subtypes in situ. Gastroenterology.

[38] Zheng GXY (2017). Massively parallel digital transcriptional profiling of single cells. Nat Commun.

[39] Wang Z (2022). Celda: a Bayesian model to perform co-clustering of genes into modules and cells into subpopulations using single-cell RNA-seq data. NAR Genom Bioinform.

[40] McGinnis CS (2019). DoubletFinder: doublet detection in single-cell RNA sequencing data using artificial nearest neighbors. Cell Syst.

[41] Hao Y (2021). Integrated analysis of multimodal single-cell data. Cell.

[42] Trapnell C (2014). The dynamics and regulators of cell fate decisions are revealed by pseudotemporal ordering of single cells. Nat Biotechnol.

[43] Qiu X (2017). Single-cell mRNA quantification and differential analysis with Census. Nat Methods.

[44] Wu T (2021). clusterProfiler 4.0: a universal enrichment tool for interpreting omics data. Innovation (Camb).

[45] Yu G (2012). clusterProfiler: an R package for comparing biological themes among gene clusters. OMICS.

