# Supplementary material for: Single-nucleus RNA sequencing reveals transcriptional heterogeneity in the blastema of favorable-histology Wilms tumor
Source: JCI Insight. 2026 Jun 9;11(15):e188078. doi: 10.1172/jci.insight.188078 (PMC13463612; doi:10.1172/jci.insight.188078)
Supplement: Supplementary file 1 [file jciinsight-11-188078-s083.pdf]

Supplemental Table S1. Cell types isolated in single nuclei RNA-seq - percent (number of nuclei)

Sample	Blastema	Epithelial	Interstitial
Tumor 1 (T1)	87% (7041)	9% (723)	4% (284)
Tumor 2 (T2)	79% (5879)	18% (1314)	3% (202)
Tumor 3 (T3)	65% (6086)	17% (1555)	19% (1775)
Tumor 4 (T4)	53% (3794)	39% (2781)	8% (561)
Tumor 5 (T5)	81% (5820)	5% (335)	14% (1002)
Tumor 6 (T6)	48% (4454)	41% (3816)	11% (1024)
Tumor 7 (T7)	91% (8911)	8% (806)	1% (105)
Tumor 8 (T8)	74% (4915)	23% (1504)	3% (212)
Tumor 9 (T9)	87% (3791)	4% (167)	9% (378)

Supplemental Table S2. Cell type by histological classification - percent

Sample	Blastema	Epithelial	Interstitial
Tumor 1 (T1)	5%	5%	90%
Tumor 2 (T2)	80%	5%	15%
Tumor 3 (T3)	90%	6%	4%
Tumor 4 (T4)	1%	90%	9%
Tumor 5 (T5)	25%	25%	50%
Tumor 6 (T6)	40%	35%	25%
Tumor 7 (T7)	95%	1%	4%
Tumor 8 (T8)	65%	30%	5%
Tumor 9 (T9)	60%	5%	35%
